# Addiction, Anhedonia, and Comorbid Mood Disorder. A Narrative Review

**DOI:** 10.3389/fpsyt.2019.00311

**Published:** 2019-05-22

**Authors:** Marianne Destoop, Manuel Morrens, Violette Coppens, Geert Dom

**Affiliations:** ^1^Collaborative Antwerp Psychiatric Research Institute (CAPRI), Faculty of Medicine and Health Sciences, University of Antwerp, Antwerp, Belgium; ^2^Psychiatric Hospital Multiversum, Campus Alexianen, Boechout, Belgium; ^3^University Department of Psychiatry, Campus Duffel, Duffel, Belgium

**Keywords:** anhedonia, disorders in the use of substances, substance abuse, addiction, depression, mood disorder, gambling, internet gaming

## Abstract

**Background:** Recently, anhedonia has been recognized as an important Research Domain Criterion (RDoC) by the National Institute of Mental Health. Anhedonia is proposed to play an essential role in the pathogenies of both addictive and mood disorders, and possibly their co-occurrence with a single individual. However, up to now, comprehensive information about anhedonia concerning its underlying neurobiological circuitries, the neurocognitive correlates, and their role in addiction, mood disorder, and comorbidity remains scarce.

**Aim:** In this literature review of human studies, we bring together the current state of knowledge with respect to anhedonia in its relationship with disorders in the use of substances (DUS) and the comorbidity with mood disorders.

**Method:** A PubMed search was conducted using the following search terms: (Anhedonia OR Reward Deficiency) AND ((Drug Dependence OR Abuse) OR Alcohol OR Nicotine OR Addiction OR Gambling OR (Internet Gaming)). Thirty-two articles were included in the review.

**Results:** Anhedonia is associated with substance use disorders, and their severity is especially prominent in DUS with comorbid depression. Anhedonia may be both a trait and a state dimension in its relation to DUS and tends to impact DUS treatment outcome negatively.

## Introduction

Disorders in the use of substances (DUS) as defined by the *Diagnostic and Statistical Manual of Mental Disorder-5 (DSM-5)* are a set of highly prevalent disorders with an enormous negative impact on individuals, their families, and society as a whole (1). From a neuroscientific perspective, DUS can be conceptualized as complex disorders, i.e., multiple symptom clusters and underlying neurobiological circuitries/systems play a role. In its core lay both a hypersensitivity to drug-related stimuli and an impairment in (executive) control over these impulses. On the other hand, and increasingly as the disorder progresses, a “darker” side has been suggested where an increase of brain-stress system, impaired stress tolerance, negative affect, and anhedonia take the upper hand (2).

From a clinical perspective, anhedonia, i.e., a markedly diminished interest or pleasure in activities that are naturally rewarding, is an essential characteristic for many addicted individuals. Anhedonia-like symptoms have been reported in the context of active chronic substance use, (protracted) withdrawal, and during sustained abstinence. Also, anhedonia may, for some individuals, act as a pre-existing vulnerability for substance initiation, regular use, and the subsequent development transition to addiction (3). The symptoms characterizing anhedonia may reflect underlying neurochemical changes, typically associated with the “dark side” of addiction, where negative reinforcement drives continuing substance use and the neurochemical picture is dominated by dysregulation of brain-stress systems (2). These may also include peripheric inflammation processes that have been reported in the context of chronic substance use and associated with depression and anhedonia (4). In line with this are the recent findings indicating that antidepressants, i.e., agomelatine, might affect anhedonia, possibly *via* decreasing C-reactive protein and increasing BDNF serum levels (5–7). Furthermore, anhedonia may have specific clinical importance, i.e., for outcome and treatment response. Indeed, anhedonia increases the likelihood of relapse and is associated with craving (3).

Characteristic of DUS is the high prevalence of comorbidity with other psychiatric disorders. This might be the result of the diagnostic vagueness inherent to the currently used diagnostic categorical systems such as DSM and ICD. Alternatively, common underlying factors may drive different behavioral–phenotypical presentations that when diagnosed “categorical” on a behavioral level results in statistical high levels of comorbidity (8). Disorders of mood (MD) are one of the psychiatric disorders that have been reported to co-occur frequently with DUS are mood disorders (MD). The co-occurrence of MD and DUS has been well established with an estimated two- to fivefold increase in odds of having an MD when the other condition is present (9). With respect to the pathogenesis of psychiatric disorders, anhedonia has been considered as a principal, transdiagnostic characteristic, within the phenotypic concept of different mental disorders, e.g., mood disorders, schizophrenia, and also DUS (10). Recent studies suggest that reward hyposensitivity within unipolar depression will be most strongly associated with a state of anhedonia characterized by motivational versus hedonic deficits (11, 12). From this perspective, it might be hypothesized that anhedonia as an underlying neurobiological construct acts as a driver explaining the high prevalence of the DUS–depression comorbidity. Alternatively, anhedonia might be a symptom within both disorders but of which its origin is based on different pathogenetic pathways, e.g., anhedonia as a result of down-regulation of reward pathways in a response of chronic substance (ab)use.

Anhedonia is by far not the only common construct underlying comorbidities between DUS and other psychiatric disorders. Indeed, using the Research Domain Criterion (RDoC) terminology, deficits in threat-related processes (Negative Valence Systems), executive control (Arousal/Regulatory Systems), and working memory (Cognitive Systems) are observed across many psychiatric disorders in both the “internalizing” spectrum (e.g., depression, anxiety) and the “externalizing” spectrum, i.e., DUS (8, 11). However, up to now, the role of anhedonia in both the pathogenesis of addiction and in the comorbidity with mood disorders has been mainly left understudied. This is an essential caveat since an increasing number of studies indicate that anhedonia, e.g., within the context of depression, is a factor that negatively impacts treatment outcome. Indeed, anhedonia is a predictor of poor longitudinal course of symptoms of major depression, suicidality, and suicidal ideation and poor response on pharmacological treatment (13–16).

Within the scope of this review, we first present ideas on conceptualizing and assessing anhedonia. Next, we review the literature exploring the relationship between anhedonia and substance use disorders. In the discussion, we extend on how these findings match with current concepts on anhedonia and how this, potentially, reflect on treatment and future research.

## Conceptualization of Anhedonia

Anhedonia refers to a decreased interest or pleasure in response to stimuli that are either by nature or previously perceived as rewarding. As such, anhedonia is inherently associated with reward processing. Reward processing involves multiple components that can be dissected experimentally in animal models but are likely intermingled in real life-situations: sensory detection of a stimulus, affective hedonic reaction, pleasure itself (liking), motivation to obtain the reward and work for it (wanting or incentive salience), and reward-related learning processes (17).

At least two broad dimensions underlying anhedonia have been identified through animal and human research: 1) reward hyposensitivity and 2) reduced approach motivation. Of importance, both aspects can be dissected regarding their underlying neurobiological pathways and neurochemical hallmarks (11).

Reward hyposensitivity has been suggested to be associated with the functionalities related to the “consummatory” part of reward processing, i.e., often reflected by the term “liking.” Pleasure experience is suggested to be mediated by the endogenous opioid and endocannabinoid receptor pathways in different brain areas (18). This component could be called the hedonic dimension of anhedonia, i.e., “hedonic anhedonia.”

Approach motivation is viewed as the driver that facilitates approach or goal-directed behavior to obtain rewards. Information encoded by dopaminergic transmission within the mesolimbic system is suggested to play a role in reward motivational value and motivational salience (17). The primary system is proposed to be dopaminergic frontostriatal circuitries. Reducing dopaminergic functioning has an adverse effect on the motivation to pursue and work for rewarding stimuli. This dimension could be called the motivational component of anhedonia, i.e., “motivational anhedonia.” Of interest, administration of a dopamine agonist (d-amfetamine) produces an increase in the willingness to work for rewards in animal models (11, 19).

**Figure 1:**
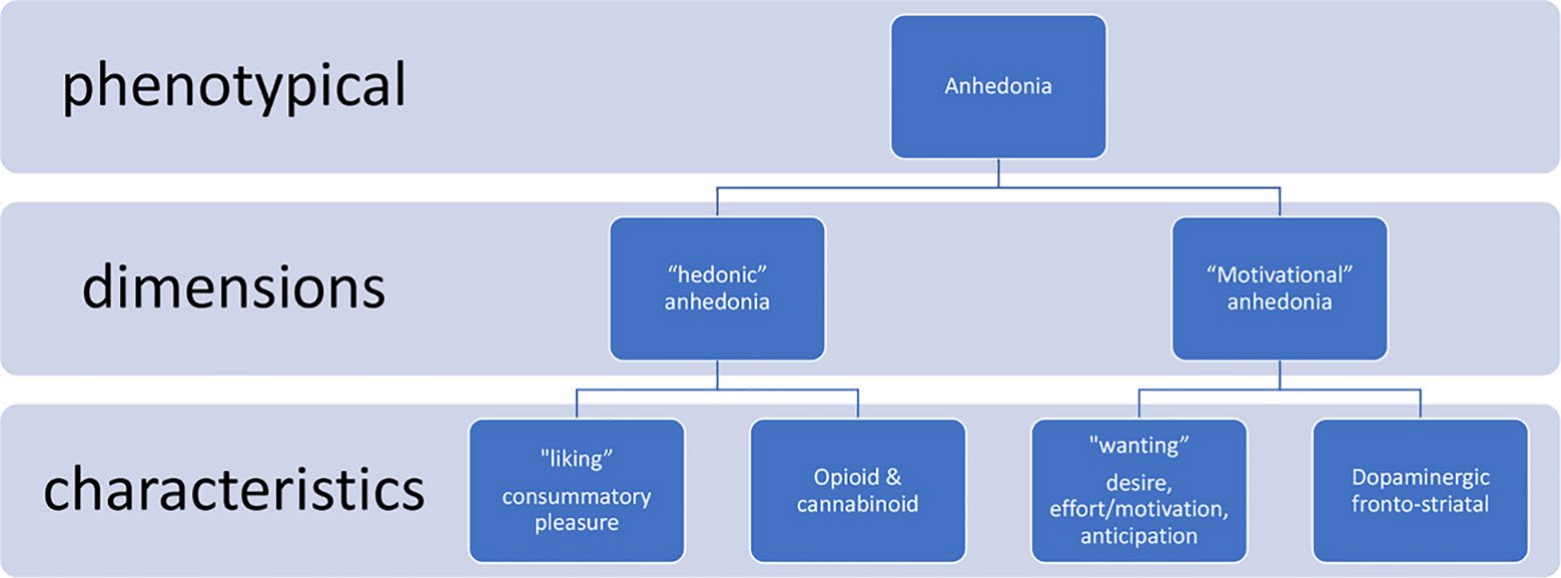
Anhedonia dimensions (11, 18).

Taken together, growing evidence from self-report, behavioral, and neurophysiological studies suggest that reward hyposensitivity and reduced approach motivation reflect anhedonia (11). From this perspective, two distinct neural circuits underlying motivational (anticipation, wanting; i.e., associated with dopamine signaling within the frontostriatal circuitry) versus hedonic (consumption, liking; i.e., associated with endogenous opioids signaling) reward-related states can be hypothesized (11). For this review, we conceptualize anhedonia to these two basic dimensions (**Figure 1**).

## Review: Aim and Questions

Within the scope of this explorative–narrative review part of this manuscript, we aim to explore the following questions:

- What is the prevalence of anhedonia within human DUS individuals?
- What types of measurement instruments of anhedonia are used in human studies within DUS samples?
- Is there a differentiation according to hedonic versus motivation anhedonia?
- How does anhedonia relate to DUS–depression comorbidity?
- What is the role of anhedonia in DUS course and treatment response?

## Method

The most recent systematic review on the relation between substance use disorders (SUD) and anhedonia reviewed the literature up to 23 May 2013 (3). So, with this review, we aimed at expanding this body of work by reviewing the literature published after this date, i.e., last 5 years. A search was performed in PubMed using the same search terms as in this latter publication (3). We included pathological gambling and internet gaming in this search because they recently were included in the DUS chapter of the *DSM-5* (and will be in the next ICD11) as addictive disorders.

**Figure 2:**
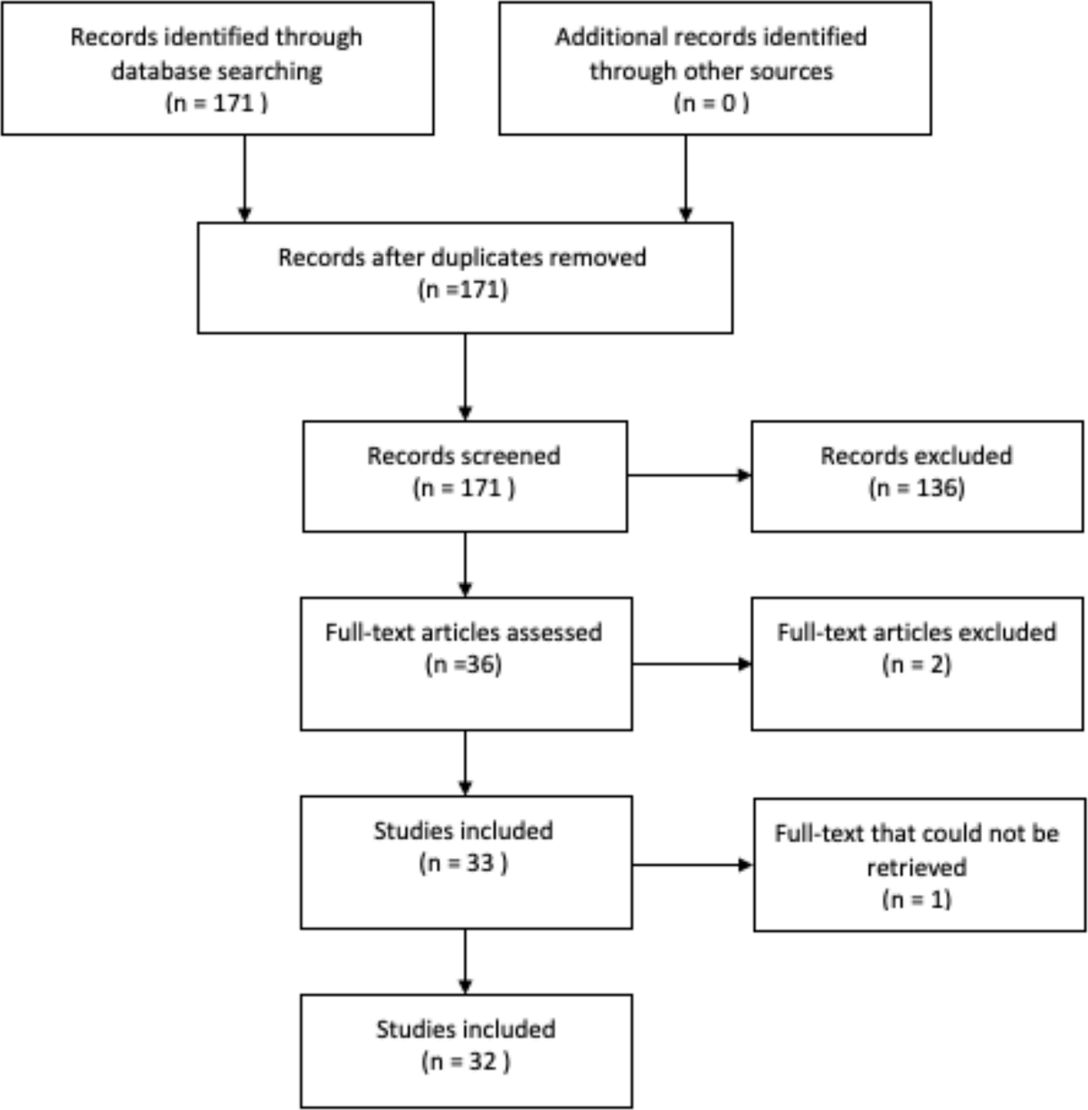
Search strategy for research papers in PubMed.

In order to obtain original studies investigating the link between anhedonia and DUS, a PubMed search (May 2013–November 2018) for English language articles was conducted using the following search terms: (Anhedonia OR Reward Deficiency) AND ((Drug Dependence OR Abuse) OR Alcohol OR Nicotine OR Addiction OR Gambling OR (Internet Gaming)). The papers were filtered for human studies only. An overview of the inclusion process can be found in **Figure 2**. The PubMed search yielded 171 results; abstract screening led to the exclusion of 136 papers, leaving 35 papers. Of these, one full paper could not be retrieved, and two validation studies were excluded, so 32 articles were included in the review.

## Results

The majority of studies (*n* = 13) focused on tobacco smoking compared to alcohol (*n* = 4), cannabis (*n* = 4), cocaine (*n* = 5), benzodiazepines (*n* = 1), and opioids (*n* = 4). Behavioral addictions remain poorly studied, i.e., one study on gambling and none on online gaming. See **Table 1** for an overview of all studies.

**Table 1:** Results from the literature review.

	Author	Sample	Anhedonia measure			Comorbidity	Result
			Self-report	Behavioral task	Neuro-biologic		
Alcohol	(20)	MDD (*n* = 4,339) MDD+AUD (*n* = 413)	MINI	/	/	MDD	Anhedonia is associated with alcohol abuse
	(21)	Trauma-exposed US military veterans (*n* = 913)	PCL-5	/	/	PTSD symptoms	Anhedonia associated with past-year alcohol consequences
	(22)	18- to 25-year Hispanic emerging adults (*n* = 181)	CES-D	/	/	/	Higher levels of anhedonia were associated with higher alcohol use severity
	(23)	College students (18–22 years) (*n* = 820)	MASQ-SF	/	fMRI while participants completed a card-guessing task, which elicits ventral striatum reactivity	/	Reduced ventral striatum reactivity to reward is associated with increased risk for anhedonia in individuals exposed to early life stress. Such stress-related anhedonia is associated with problematic alcohol use
Nicotine	(24)	Non-daily cigarette smokers (18–24 years) (*n* = 518): smoking more than 1/m more than 6m	SHAPS online after 3, 6, and 9 months follow-up	/	/	/	Anhedonia is not predictive for other tobacco products use (OTP), but those with anhedonia used hookah more frequently
	(25	Adults in a smoking cessation clinical trial (*n* = 1,122), min 10 sig/d min 6 m: placebo (*n* = 131), bupropion (*n* = 401), or NRT (*n* = 590)	Ecological momentary assessments 4 times a day 5 days prior and 10 days after target quit day	/	/	/	Anhedonia is associated with dependence and was suppressed by agonist administration
	(26)	Ninth-grade students (13–15 years) (*n* = 3299): ever-smokers (*n* = 343), never-smokers (*n* = 2,956)	SHAPS	/	/	/	Anhedonia is associated with smoking initiation in the overall sample and higher initiation susceptibility in the subsample never-smokers
	(27)	Non-treatment-seeking smokers (more than 10 sig/d, more than 2 years) (*n* = 125) attending 2 counterbalanced experimental sessions (abstinent (for 16 h) vs. none abstinent)	SHAPS	Picture Rating Task	/	/	Greater anhedonia associated with less negative affective reactivity to negative pictures
	(28)	Smoking participants in a double-blind cessation clinical trial (*n* = 1,236): nicotine patch (*n* = 216), nicotine lozenge (*n* = 211), bupropion (*n* = 213), patch + lozenge (*n* = 228), bupropion + lozenge (*n* = 221), placebo (*n* = 147)	Ecological momentary assessments 4 times a day 5 days prior and 10 days after target quit day	/	/	/	High craving anhedonia group reported higher dependence, were less likely to have received combination nicotine replacement, reported lower week 8 abstinence rates and relapsed sooner
	(29)	Smokers (more than 5 sig/d) (*n* = 1125)	MASQ-S	/	/	/	Urgency is associated with smoking at average or higher levels of anhedonia; it was unrelated to smoking when few anhedonia symptoms were endorsed
	(30)	Adult smokers (*n* = 525) (more than 10/d) from a cessation clinical trial on 21 mg/day nicotine patch therapy during 8 weeks	SHAPS	/	/	/	70 participants (13%) were anhedonic, men were more anhedonic, anhedonic smokers were more likely to be abstinent
	(31)	Ninth-grade students (*n* = 807): 294 no history of SUD, 166 lifetime history of drug/alcohol use without tobacco, 115 lifetime history of drug/alcohol use with tobacco	SHAPS	/	/	/	Teens with lifetime alcohol/drug use without tobacco had higher anhedonia
	(32)	Ninth-grade students (*n* = 3,310): 2,557 neither conventional nor e-cigarettes, 412 e-cigarettes only, 152 conventional cigarettes only, 189 conventional and e-cigarettes	SHAPS	/	/	/	Anhedonia was higher in e-cigarette only vs. non-users. An ordered effect of dual-use vs. e-cigarette use only vs. non-use was found for anhedonia
	(33)	Veterans with MDD or dysthymia (*n* = 80): 36 depressed smokers and 44 depressed non-smokers	MASQ-S BIS/BAS	Probabilistic reward task that measures reward-learning	/	MDD-dysthymia	Depressed smokers reported higher trait anhedonia and reduced BAS reward responsiveness compared to non-smokers. Depressed smokers demonstrated greater acquisition of reward-based learning
	(34)	Adults from smoking cessation clinical trial (*n* = 1,175) (min 10 sig/d last 6 months): bupropion, nicotine lozenge, nicotine patch + lozenge, bupropion + nicotine lozenge or placebo	Ecological momentary assessments 4 times a day from 5 days prior to 10 days after target quit day	/	/	/	Anhedonia showed an inverted U- pattern of change in response to tobacco cessation and was associated with the severity of withdrawal symptoms and tobacco dependence. Post-quit anhedonia was associated with decreased latency to relapse and with lower 8-week point prevalence abstinence. NRT suppressed the increase in abstinence-related anhedonia
	(35)	Adults recruited *via* announcements (*n* = 275) (more than 10 sig/d): participants attended a baseline visit that involved anhedonia followed by 2 counterbalanced visits after 16 h smoking abstinence and non-abstinent	SHS TEPS CAI	Behavioral smoking task measuring relative reward value of smoking	/	/	Higher anhedonia predicted quicker smoking initiation and more cigarettes purchased, partially mediated by low and high negative mood states. Abstinence amplified the extent to which anhedonia predicted cigarette consumption among those who responded to the abstinence manipulation, but not the entire sample
	(36)	Smokers enrolled in a smoking cessation treatment study (*n* = 1,469) (more than 10 sig/d more than 6 m): bupropion (*n* = 264), nicotine lozenge (*n* = 260), nicotine patch (*n* = 262), bupropion + lozenge (*n* = 262), placebo (*n* = 189)	Life time anhedonia *via* CIDI	/	/	depression	Anhedonia predicted cessation outcome
Cannabis	(37)	Cannabis users between 15 and 24 years (*n* = 162): 47 early onset, before 16 years; 115 late onset	Online OLIFE	/	/	Schizotypy	Early-onset cannabis use is associated with higher levels of anhedonia in females only
	(32)	Student at the age of 14 (*n* = 3,394) at baseline, 6-, 12-, and 18-month follow-up	SHAPS	/	/	/	Anhedonia is associated with subsequent marijuana use escalation amplified by cannabis-using friends, but baseline marijuana use is not related to the rate of change in anhedonia.
	(38)	20-year-old men (*n* = 158), recruited at the age of 6–17 m	SHAPS	/	fMRI during a 24-trial slow event-related card-guessing game that assesses response to anticipation and receipt of monetary reward		The escalating trajectory group displayed a pattern of negative NAcc–mPFC connectivity that was linked to higher levels of anhedonia
	(39)	MDD subgroup from a national survey (*n* = 2,348): users with CUD vs. users without CUD	*DSM-IV* criteria	/	/	MDD	Level of cannabis use is associated with anhedonia
Stimulants	(19)	Treatment-seeking adults with cocaine dependence: on contingency management (*n* = 85): 40 placebo, 45 levodopa	SHAPS	PR task	/	/	L-dopa did not improve outcomes of CM, nor was the effect moderated by anhedonia; anhedonia may be a modifiable individual difference associated with poorer outcome of CM
	(40)	CUD participants (*n* = 46)	CSSA	/	RewP of ERP	/	RewP is correlated with anhedonia, and anhedonia explained a significant amount of variance in the RewP amplitude
	(41)	Current cocaine abusers (*n* = 23) and participants with no drug history (*n* = 24)	SHAPS	/	ERP after reward receipt	/	Anhedonia is associated with reward motivation, diminished reward feedback, and diminished monitoring
	(42)	Current cocaine abusers, outpatients (*n* = 23) and controls with no drug history (*n* = 27)	SHAPS CPCSAS	/	Go/NoGo task while EEG was recorded. Valenced pictures from the International Affective Picture System	/	Cocaine users performed more poorly than controls on the inhibitory control task. Cocaine users were more anhedonic. Higher levels of anhedonia were associated with more severe substance use. Inhibitor control and anhedonia were correlated only in controls
	(43)	Cocaine-dependent patients, free from cocaine during the last 3 weeks (*n* = 23) and healthy controls (*n* = 38)	Chapman psychosis-proneness scales (with revised physical anhedonia and revised social anhedonia)	/	A paired-stimulus paradigm to elicit three mid-latency auditory evoked responses (MLAER), namely, P50, N100, and P200	Psychosis proneness	Social anhedonia scores accounted for the largest proportion of variance in P200 gating. Poorer P50 gating is related to higher scores on the social anhedonia scale in healthy controls and across mixed samples of cocaine-dependent patients
Opioids	(44)	Heroin-dependent participants on opioid maintenance (*n* = 90): on methadone (*n* = 55) or on buprenorphine (*n* = 35); and recently abstinent (up to 12 months) opioid-dependent participants (*n* = 31); and healthy controls (*n* = 33)	SHAPS TEPS	/	/	/	Elevation in anhedonia in opioid-dependent participants
	(45)	Patients (mostly inpatients) with opioid dependence (*n* = 306): 1,000 mg naltrexone implant + oral placebo (*n* = 102), placebo implant and 50 mg oral naltrexone (*n* = 102), both placebo (*n* = 102)	FAS CSPSA	/	/	/	Anhedonia was elevated at baseline and reduced to normal within the first 1–2 months for patients who remained in treatment and did not relapse, no difference between groups
	(46)	Opioid-dependent patients 10–14 days after withdrawal (PODP) (*n* = 36) and healthy controls (*n* = 10)	SHAPS	Affect-modulated startle response (AMSR)	Cue reactivity task during which participant’s RPFC and VLPFC were monitored with functional near-infrared spectroscopy	/	PODP reported greater anhedonia on self-report, reduced hedonic response to positive stimuli in the AMSR task, reduced bilateral RPFC and left VLPFC activity to food images and reduced left VLPFC to positive social situations compared to controls. Patients with anhedonia showed reduced response to positive social stimuli and food
	(47)	Detoxified heroin-dependent patients recruited from addiction-treatment centers (*n* = 12) 2 weeks after detoxification starting extended-release naltrexone (XRNT) and healthy subjects (*n* = 11)	SHAPS	/	[^123^I]FP-CIT SPECT-scan imaging striatal DAT binding: 1 before and 1 2 weeks after injection with XRNT	/	XRNT does not affect anhedonia, but with a significant reduction of depressive symptoms
Gambling	(48)	Outpatients with Parkinson’s disease (*n* = 154): 34 fulfilled criteria for impulse control disease (ICD), of which 11 met criteria of pathological gambling (PG)	SHAPS	/	/	Parkinson’s disease	PG had a higher incidence of anhedonia
Internet gaming		/	/	/	/	/	/
Benzodiazepines	(49)	MDD outpatients of the MDPU database (Mood Disorder Psychopharmacology Unit) (*n* = 326): 79 benzodiazepine users, 247 nonbenzodiazepine users	MADRS	/	/	MDD	Anhedonia was greater in the benzodiazepine group, and anhedonia was the strongest predictor of regular benzodiazepine use

OLIFE, Oxford-125 Liverpool Inventory of Feeling and Experiences; PCL-5 = PTSD Checklist for DSM-5; SHAPS, Snaith–Hamilton Pleasure Scale; TEPS, Temporal Experience of Pleasure Scale; REI, Rewarding Events Inventory; CES-D, Center Epidemiological Studies Depression Scale; PR task, progressive ratio task; FAS, Ferguson Anhedonia Scale; CSPSA, Chapman Scale of Physical and Social anhedonia; MASQ-S, Mood and Anxiety Symptoms Questionnaire-Short Form (with Anhedonic Depression subscale); CSSA, Cocaine Selective Severity Assessment Scale; RewP, Reward Positivity component; TEPS, Temporal Experience of Pleasure Scale; BIS/BAS, Behavioral Inhibition/Behavioral Activation Scale; CPCSAS, Chapman Physical and Chapman Social Anhedonia Scales; SHS, Subjective Happiness Scale; CAI, Composite Anhedonia Index; MINI, Mini International Neuropsychiatric Interview; CIDI, Composite International Diagnostic Interview; MADRS, Montgomery–Asberg Depression Rating Scale.

### Types of Measures of Anhedonia Used Within DUS Studies

Self-report measures were, by far, the most used instruments, i.e., all studies included self-report measures. Of these, the Snaith–Hamilton Pleasure Scale (SHAPS) (50) was most frequently used, i.e., in 15 of the 32 studies. Within the depression research, the SHAPS has been validated and remains the gold standard. It measures the consummatory pleasure (51) typically. However, given the recommendation that any scale should be validated in the population of interest prior to use, it needs to be noted that none of the self-report scales found in this review was ever validated within DUS populations. This particularly warrants interpretation of the current results.

Of interest, three studies used ecological momentary assessments (EMAs) during four times a day in a smoking cessation trial (25, 28, 34). It was questioned how much pleasure the participants experienced during the day on three domains (social, recreation, and performance/accomplishment). EMA might be a promising methodology providing data better covering the actual evolution of symptoms than (retrospective) self-report and is increasingly used in both depression and addiction research (52, 53). However, as yet, no validated set of EMA-implementable questions on anhedonia have been developed.

Few studies (*n* = 4) used behavioral tasks. Guillot et al. used the Picture Rating Task, which is a measure of affective valence related to positive, negative, and smoking cues (27). In this task, participants were instructed to rate the pleasantness of each stimulus by pressing keys corresponding to seven-point Likert scale from −3 (very unpleasant) to 3 (very pleasant). Positive, negative, smoking, and neutral images are shown. In this task, anhedonia has been inversely related to pleasantness ratings of positive or reward-related stimuli.

Liverant et al. (33) used a signal detection task designed to assess modulation of behavior in response to rewards, which was already used in trials with MDD and bipolar disorders (54). In the latter studies, an inverse relationship between response bias and anhedonia was already demonstrated.

Leventhal et al. used a behavioral task measuring the relative reward value of smoking (36). This task yields objective behavioral measures of the relative value of a) initiating smoking versus delaying smoking for money and b) self-administering cigarettes for money when given the opportunity to smoke.

Wardle et al. used a progressive ratio procedure as a behavioral measure of anhedonia (19). Participants can choose two options in which option A results in greater rewards in exchange for greater effort while option C results in less reward but requires less effort. Fewer key presses for A indicates motivational anhedonia. It has to be noted that this type of behavioral measure is not strongly related to the SHAPS (55).

Taken together, the four studies using behavioral tasks all used a different paradigm. It remains unclear as to which aspect/dimension of anhedonia they tap in and how they relate with self-reported anhedonia.

Seven studies used neurobiological, i.e., neurophysiological or imaging, measures of anhedonia. First, an functional magnetic resconance imaging (fMRI) study in young cannabis users implemented a two-card guessing game that assessed response to anticipation and receipt of monetary reward (38). In this paradigm, anhedonia was associated with a pattern of negative Nucleus Accumbens (NAcc)–medial Prefrontal Cortex (mPFC) connectivity.

Parvaz et al. used a gambling task predicting whether they would win or lose money on each trial, while ERP data were required (40). Reward Positivity component (RewP) in response to predicted win trials was extracted from the ERPs. RewP is attributed to the same brain regions that are also implicated in anhedonia (i.e., ventral striatum and mPFC). The results showed that RewP amplitude in response to rewarded trials correlated with anhedonia severity in CUD.

Morie et al. performed two ERP studies in cocaine abusers and healthy controls (41, 42). In Morie et al. (41), a speeded response task with varying probabilities of reward is used. Cocaine users showed blunted response to reward-predictive cues and to feedback about task success or failure. Anhedonia measured by the SHAPS was also associated with diminished monitoring and reward feedback in cocaine users. The measures of anhedonia were associated with reward motivation in both cocaine users and healthy controls (41). Morie et al. (42) used a Go/NoGo task in response to valenced pictures. Though this is more a measure for executive functioning, i.e., inhibition and performance monitoring, a correlation was found between inhibitory control and anhedonia, but only in controls.

In a small group of detoxified heroin-dependent patients, striatal dopamine transporter binding was assessed by [^123^I]FP-CIT single photon emission computed tomography (SPECT) before and 2 weeks after injection with extended-release naltrexone (47). Although depression scores were higher for patients at baseline and depression scores were lower after extended-release naltrexone (XRNT) treatment, no associations could be found for anhedonia.

Finally, a large fMRI study with 820 college students used a ventral striatum reactivity task, a blocked number-guessing paradigm, consisting of three blocks of positive feedback, three blocks of negative feedback, and three control blocks (23). Reduced ventral striatum reactivity to reward is associated with increased risk for anhedonia in individuals exposed to early life stress. This interaction is linked to other depressive symptoms and problematic alcohol use.

In only one study were self-report, behavioral, and neurobiological measures combined (46). Thirty-six opioid-dependent patients and 10 healthy controls filled in the SHAPS and performed the affect-modulated startle response (AMSR), a psychophysiological measure of emotional valence, that was used before to assess hedonic responses to standardized reward-related stimuli. Four categories of stimuli can be derived: positive, negative, neutral, and drug-related. Meanwhile, acoustic startle probes were presented at variable points and the eye-blink component of the startle reflex was recorded by EMG. All participants completed a standard visual cue activity paradigm while being monitored with functional near-infrared spectroscopy (fNIRS). Stimuli consisted of three hedonically positive categories (highly palatable food, positive social interaction, and emotional intimacy) as well as emotionally neutral stimuli. Opioid-dependent patients reported greater anhedonia on self-report, reduced hedonic response to positive stimuli in the AMSR task, and reduced bilateral RPFC and left VLPFC to food imaged and reduced left VLPFC to positive social situations compared to controls.

Taken together, although more studies used a neurobiological measure as compared to behavioral task only, again all of them used a different paradigm, making a comparison of the results difficult. Also, it remains de be defined what dimensions/aspects of anhedonia are captured by these different paradigms, although some studies provide indications for the motivational component (e.g., fronto-striatal connectivity).

### Anhedonia Within DUS Populations

Very few studies compared anhedonia between a sample of DUS patients with non-DUS controls. Other studies focused on the relationship between substance abuse and severity-related variables in relation with anhedonia in samples of DUS individuals.

Studies with a healthy control group showed consistently that cocaine abusers, heroin-dependent individuals, and benzodiazepine-dependent individuals were more anhedonic versus controls. Also, higher levels of anhedonia associated with more severe substance use (42, 44, 46, 47, 49).

Studies within DUS samples without control revealed a similar result; i.e., anhedonia was associated with substance use variables. Three studies on alcohol showed a positive association between anhedonia and alcohol use severity and related consequences (20–22). Within cigarette smokers, most studies provide indications of an adverse effect of anhedonia on smoking: initiation, smoking susceptibility, and severity (24, 26, 29, 35). Finally, early onset of cannabis use, subsequent escalation of marijuana use, and level of use have been associated with higher levels of anhedonia (32, 37, 39). One study on gambling showed higher levels of anhedonia in a gambling subsample of Parkinson’s disease patients (48). However, this study included only 11 gamblers, warranting careful interpretation.

Taken together, across different substances, indications are consistent that 1) DUS individuals have higher levels of anhedonia than controls and that 2) anhedonia might be related with early onset of substance use and subsequent severity of DUS.

### Time Course of Anhedonia: Trait or State?

For nicotine-dependent individuals, there is evidence that anhedonia is both a state and a trait factor. First, in a longitudinal study with 518 young participants, the presence of anhedonia predicted the use of hookah (24). Evidence for anhedonia as a trait can also be found in the study of Leventhal (36), which is already described above (36). The trait anhedonia predicted quicker smoking initiation and more cigarettes purchased, and 16-h smoking abstinence amplified the extent to which anhedonia predicted cigarette consumption. In addition, a recent study showed that 1) anhedonia is associated with smoking initiation and 2) adolescents with higher (vs. lower) anhedonia who have never tried smoking may be more susceptible to smoking initiation perhaps due to stronger pro-smoking intentions or willingness to smoke (26).

Data supporting trait anhedonia for other substances are few. For cannabis, anhedonia has been associated with both early onset of cannabis use and marijuana use escalation in early adolescence (32, 37).

On the other hand, anhedonia can be a part of smoking withdrawal. Cook et al. (34) demonstrated an inverted U-pattern in response to tobacco cessation, which was associated with the severity of withdrawal symptoms and tobacco dependence (34). In the 6-month follow-up study with opioid-dependent patients (mostly inpatients), elevated anhedonia levels at baseline reduced to normal after 1 to 2 months for patients who did not relapse (45). In the study of Garfield et al. (44), elevation of anhedonia was found in opioid-dependent participants compared to healthy controls (44). Among participants on opioid pharmacotherapy (i.e., methadone and buprenorphine), a significant association was found between the frequency of recent illicit opioid use and anhedonia scores, which supports the hypothesis that opioids can cause anhedonia. On the other hand, no association was found between duration of abstinence and anhedonia in the group of abstinent opioid-dependent participants.

### Anhedonia and DUS and Depression Comorbidity

Two out of four studies concerning alcohol use disorder (AUD) focused on comorbidity as well. In an major depressive disorder (MDD)-subsample of the Mental Health in the General Population (MHGP), 4,339 subjects met the criteria for MDD (20). In the MDD population, 413 AUD subjects were identified, including 138 subjects with alcohol abuse and 275 with alcohol dependence. Anhedonia was associated with alcohol abuse in the group with MDD and AUD compared to the group without AUD (OR 1.66).

A sample of 916 trauma-exposed US military veterans was drawn from a larger dataset from the National Health and Resilience in Veterans Study (NHRVS, 21). A subsample was chosen that endorsed a “worst” traumatic event on the Traumatic History Screen. In this nonclinical sample, associations between the seven-factor hybrid model of PTSD symptoms and alcohol consumption and consequences were found. Lifetime anhedonia, together with dysphoric arousal and negative affect, was most strongly associated with past-year alcohol consequences.

MDD comorbidity is studied in nicotine papers as well. In an MDD/dysthymia subsample of veterans from a large VA Healthcare System in the Northeast United States, 36 depressed smokers were compared to 44 depressed non-smokers (28). Depressed smokers reported more anhedonia and reduced reward responsiveness. However, on a probabilistic learning task, depressed smokers showed a stronger preference for the more frequently rewarded stimulus, which suggests that depressed smokers demonstrated more robust acquisition of reward-based learning.

Leventhal et al. (36) adjusted the relation between anhedonia and depressed mood with relapse in nicotine for lifetime depressive disorder based on the CIDI. Depressed mood did not predict cessation outcome, while anhedonia did (36).

For cannabis, only one study focused on comorbidity between CUD and MDD. Feingold et al. (39) selected an MDD subgroup from a national survey and concluded that the level of cannabis use was associated with more symptoms at follow-up, notably anhedonia, while remission rates did not differ between MDD with or without CUD (39).

Rizvi et al. (49) demonstrated that anhedonia was more significant in MDD patients using benzodiazepines, with anhedonia being the strongest predictor of regular benzodiazepine use (49).

One fMRI study showed a decreased ventral striatum reactivity to the (monetary) reward associated with an increased risk for anhedonia, especially for those participants who were exposed to early life stress (23). This might suggest that for these individuals specifically, motivational anhedonia is impaired.

### Anhedonia and Effect on Treatment of DUS

Most studies showed an adverse effect of anhedonia on treatment effect. In a large randomized, double-blind placebo-controlled smoking cessation trial, four distinct types of quit-day withdrawal were identified: the moderate withdrawal class were the least likely to report high levels of any individual symptom for hunger and anhedonia. The high-craving anhedonia group reported high levels of craving and anhedonia. The affective withdrawal group was scoring high on poor concentration and negative affect. The hunger group reported high quit-day hunger, but low on other indicators. The high-craving anhedonia group reported lower week 8 abstinence and relapsed sooner but were also less likely to have received combination nicotine replacement in this trial (28).

In another smoking cessation treatment study with 1,469 participants, lifetime anhedonia predicted increased odds of relapse after 8 weeks and 6 months (36). Moreover, post-quit anhedonia was associated with decreased latency to relapse and with lower 8-week point prevalence abstinence. Similar findings were demonstrated in the study of Piper using the same design and method (28). They reported lower abstinence after 8 weeks for the high craving anhedonia group.

Wardle et al. (19) demonstrated that anhedonia was associated with poor treatment outcome (i.e., cocaine-negative urines) for cocaine-dependent participants following contingency management. Also, a dopamine-agonist (L-DOPA) did not improve outcomes in this study, nor was the effect of L-DOPA moderated by anhedonia (19).

Only in one study did anhedonia have a positive effect on treatment (30). In the clinical cessation trial on 21-mg nicotine patch a day for 8 weeks, 70 participants were anhedonic based on the SHAPS. The anhedonic smokers were more likely to be abstinent on a nicotine patch.

## Discussion

In this exploratory–narrative review, we identified 32 original research papers exploring anhedonia and its relationship with substance use disorders. Results provide indications that 1) anhedonia is associated with substance use problems/disorders and their severity, 2) anhedonia is especially prominent in DUS with comorbid depression and early life stress experiences, 3) anhedonia may be both a trait and a state dimension in its relation to DUS, and 4) anhedonia tends to negatively impact DUS treatment outcome. Finally, most evidence points to motivational anhedonia as the most involved subdimension of anhedonia within its relationship with DUS.

Overall, the findings in this review, focusing on articles over the last 5 years, are in line with the earlier review of Garfield et al. (3). Across the different substances of abuse, findings in this review provide indications that anhedonia—as a broad concept—is associated with DUS and DUS severity. However, these findings need to be looked upon prudently. Indeed, the number of studies using a control group remains very limited. Also, the severity measures used throughout the different studies are very variable, leaving consistent interpretation difficult. Altogether, the number of studies remains very limited especially when compared to the number of studies published on impulse/executive control in SUD. This is remarkable. Indeed, in a recent consensus paper, RDoC Positive Valence System (Reward Valuation, Expectancy, Action Selection, Reward Learning, Habit) was put forward as an essential domain with respect to the pathogenesis of addictive disorders, implicated in vulnerabilities for initiation, continuation, and chronicity of the disorder (8). Anhedonia can be positioned on the bridge of both negative and positive Valence Systems, but associates close to Reward Valuation, Reward Expectancy, and Reward Learning. This theoretical ground and the findings of our review indicate that anhedonia deserves more attention.

Moreover, anhedonia is looked upon as an important “transdiagnostic” concept underlying many different psychiatric disorders, e.g., depression, bipolar disorder, and schizophrenia (11). All these disorders relate, in different ways, to altered reward processing. Finally, anhedonia might have relevance bridging with a growing literature on the role of inflammation in the pathogenesis of psychiatric disorders such as mood disorders or addictive disorders (56). From this perspective, it can be hypothesized that a neurobiological vulnerability to inflammatory stimuli may drive the link between chronic substance use (early life stress) and anhedonia.

A sizable number of (large) studies in this review focused on comorbidity and provided indications that DUS patients with a comorbid mood disorder had higher levels of anhedonia as compared to single diagnosis groups. These findings give some ground for the hypothesis that anhedonia might be a common factor underlying both types of disorder or at least a subtype of each. Subtypes in depression with anhedonia being the prominent feature have recently been suggested. Specifically, an “inflammatory” subtype has been proposed with a neurobiological vulnerability to inflammatory stimuli that drive the link between stress and anhedonic symptoms (56). Of interest, early childhood adversity may be one of the most critical factors modulating this neurobiological vulnerability. It is remarkable that two studies in this review showed a clear association between anhedonia and substance use severity, specifically in a population of individuals exposed to trauma (21, 23). Given the high prevalence of early childhood adversity within individuals with DUS, future studies need to explore whether this subgroup is associated with anhedonia.

Research on anhedonia in other psychiatric disorders, e.g., depression, can also help to provide more insight into how research on anhedonia in SUD needs to be done. As mentioned above, self-reports are the most used instrument, while they are mostly unable to distinguish the different aspects of reward processing and reward learning. In depression literature, however, various aspects of reward in relation to anhedonia could be disentangled based on numerous studies combining behavioral tasks and neurobiological measures, mainly event related potential (ERP) studies. Neuroimaging studies could be useful as well, taking into account the idea that fMRI paradigms are mostly unable to dissect into anticipatory, consummatory, and learning components of reward processing (23). A multimodal approach using the same paradigms in future research projects is recommended.

Data from this review show mixed results as to the trait versus state characteristic of anhedonia within the context of substance use. Some studies give support to the hypothesis that anhedonia might be a trait that underlies a vulnerability for early substance use initiation and early escalation. This is in line with the self-medication theory whereby substances are used to mediate mood disorders or innate reward deficiencies (9). Also, adolescents with high stress and amygdala reactivity are more likely to consume a full standard alcoholic drink, are more likely to experience early intoxication, and are at a heightened risk for the onset of an alcohol use disorder (57). In line with this, anhedonia can be hypothesized as a vulnerability trait for early substance use trajectories and subsequent increase of DUS risk. A hypothesis is also in line with the reward deficiency hypothesis (58). Inversely, different studies in this review indicate that anhedonia is associated with ongoing substance use and withdrawal while improving over time in abstinence. This is in line with earlier studies showing improvement in reward responsiveness during treatment and abstinence (59). These findings are indicative of a state characteristic. However, longitudinal studies remain very scarce, i.e., in this review, only one study followed the course of anhedonia over a 6-month abstinence period showing improvement over time (45). So, any conclusion concerning trait or state is at best preliminary.

Several studies in this review showed a negative influence of anhedonia on DUS course and treatment effect, i.e., shorter posttreatment abstinence and higher relapse rates. This is confirmation of findings presented in the earlier review on this topic showing that anhedonia increases the likelihood of relapse and is associated with craving (3). In the depression research, anhedonia negatively influences disease course. This has also been documented within the context of treating depression (13–16). It can be hypothesized that anhedonia as a transdiagnostic characteristic modulates disease course and outcome.

Within the context of depression treatment, existing psychological and pharmacological treatments have proved to be rather ineffective for treating anhedonia. Some of the more commonly used antidepressants, e.g., fluoxetine, may even worsen anhedonic symptoms (60–62). Of importance, newer treatments such as ketamine are shown to have improvement of anhedonia, even in treatment-resistant depression (63, 64). This is of interest, also from the perspective of indication that ketamine can be used within the context of treatment of DUS (65). Although, at this point, no study has been published exploring the effectiveness of ketamine as a treatment for patients with DUS and depression/anhedonia comorbidity, this is an exciting idea. Of interest in this review is the finding that substitution treatment (i.e., nicotine patch) might be beneficial specifically for smokers scoring high on anhedonia. Powers et al. (30) showed an increased likelihood of short-term abstinence using a 21-mg/day nicotine patch therapy. Cook et al. (34) observed that administering nicotine replacement therapy suppressed abstinence-induced anhedonia and alleviated nicotine withdrawal symptoms during short-term abstinence. Moreover, depressed non-smokers show significant declines in depressive symptoms during nicotine patch treatment, suggesting that NRT (and nicotine patch in particular) may have antidepressant-like effects (66). It has been hypothesized that nicotine exposure ameliorates the hypoactivation in crucial structures of the reward pathway (including caudate, nucleus accumbens, putamen) among depressed smokers, with data showing increased activation after nicotine administration in the dorsal striatum during anticipatory reward responding and in the medial prefrontal cortex associated with sensitivity to reward (67). It has to be noted that the sample of anhedonic participants in the study of Powers et al. (30) was small, and the lack of a placebo condition made it difficult to draw inferences about the impact of nicotine patch therapy on pretreatment anhedonia or depression more generally. Finally, there is preliminary evidence that aripiprazole might promote alcohol abstinence and reduce anhedonia, possibly *via* dopaminergic and serotonergic modulations at the fronto-subcortical circuitries (68). However, this needs future replication.

Taken together, although anhedonia is notably challenging to treat and can negatively impact disease course, these preliminary studies hold promises for developing future—pharmacological—treatments.

Findings in this review need be looked upon critically. Several limitations need to be taken into account. First, the vast majority of studies focus on tobacco smoking. Other substances of abuse remain largely understudied, and regarding behavioral addictions, the information is zero. Next and most importantly, throughout the studies, a variety of anhedonia measures has been used. For none of these measures it is known what exact anhedonia dimension they measure, neither is enough information available on how these measures relate. This makes a comparison between studies impossible and may be responsible for sometimes contradictory findings. Third, different study designs and samples are used, which makes it difficult to draw general conclusions about the temporal and causal relationships between anhedonia and DUS. Finally, ours is an explorative, narrative review highlighting the broad field of the anhedonia–DUS relationship. Future hypothesis-driven studies are needed both on the clinical consequences and on elucidating the exact underlying mechanisms and neurocognitive dimensions.

## Conclusions

Findings from this review provide indications that anhedonia might be of relevance for a better understanding of the pathogenesis of addictive disorders and their comorbidities. Anhedonia might prove to be an unimportant transdiagnostic dimension underlying many disorders in their relationship with different reward processing impairments. Within the National Institute of Mental Health’s (NIH) Research Domain Criteria (RDoC), anhedonia is conceptualized as an RDoC Element (behavior) within the following Domains and Constructs: 1) Domain: Negative Valence Systems; 2) Construct: Loss and Construct. However, anhedonia might also be linked to other domains, i.e., Positive Valence Systems (11), so anhedonia might be important in bridging these systems and/or reflect different subgroups/mechanisms.

However, in contrast to the field of impulsivity, the study of anhedonia in the relationship with DUS is only nascent. Reflective of this is not only the relatively small number of studies but also the variability of measures and concepts used in the different studies. There is a great need of consensus in defining the neurocognitive dimensions and best measurement instruments/paradigms to help the field move on more quickly. Within this context, the recent international consensus paper identifying the most critical cognitive domains within neuroscience of addictions is a vital initiative (8). Let us see how and when anhedonia finds a place in this model.

## Author Contributions

All authors contributed to the manuscript conception design and writing.

## Conflict of Interest Statement

The authors declare that the research was conducted in the absence of any commercial or financial relationships that could be construed as a potential conflict of interest.
